# Molecular evidence for distinct modes of nutrient acquisition between visceral and neurotropic schistosomes of birds

**DOI:** 10.1038/s41598-018-37669-2

**Published:** 2019-02-04

**Authors:** Roman Leontovyč, Neil D. Young, Pasi K. Korhonen, Ross S. Hall, Jana Bulantová, Veronika Jeřábková, Martin Kašný, Robin B. Gasser, Petr Horák

**Affiliations:** 1 Department of Parasitology, Faculty of Science, Charles University, Prague, Czech Republic; 2 Department of Veterinary Biosciences, Melbourne Veterinary School, Faculty of Veterinary and Agricultural Sciences, The University of Melbourne, Parkville, Victoria, 3010 Australia

## Abstract

*Trichobilharzia* species are parasitic flatworms (called schistosomes or flukes) that cause important diseases in birds and humans, but very little is known about their molecular biology. Here, using a transcriptomics-bioinformatics-based approach, we explored molecular aspects pertaining to the nutritional requirements of *Trichobilharzia szidati* (‘visceral fluke’) and *T*. *regenti* (‘neurotropic fluke’) in their avian host. We studied the larvae of each species before they enter (cercariae) and as they migrate (schistosomules) through distinct tissues in their avian (duck) host. Cercariae of both species were enriched for pathways or molecules associated predominantly with carbohydrate metabolism, oxidative phosphorylation and translation of proteins linked to ribosome biogenesis, exosome production and/or lipid biogenesis. Schistosomules of both species were enriched for pathways or molecules associated with processes including signal transduction, cell turnover and motility, DNA replication and repair, molecular transport and/or catabolism. Comparative informatic analyses identified molecular repertoires (within, e.g., peptidases and secretory proteins) in schistosomules that can broadly degrade macromolecules in both *T. szidati and T. regenti*, and others that are tailored to each species to selectively acquire nutrients from particular tissues through which it migrates. Thus, this study provides molecular evidence for distinct modes of nutrient acquisition between the visceral and neurotropic flukes of birds.

## Introduction

Parasitic flatworms of the family Schistosomatidae have complex biologies and life histories^1,2^, and are usually significant pathogens^3^. Their life cycles involve aquatic snails as intermediate hosts, in which asexual reproduction takes place, and vertebrates such as mammals and birds as definitive hosts, in which sexual reproduction occurs^1,2^.

On one hand, members of the genus *Schistosoma* infect humans and/or other mammals as definitive hosts. In humans, *Schistosoma mansoni*, *S. japonicum* and *S. haematobium* are the three predominant species and are significant pathogens in their own right. They affect ~230 million people worldwide and cause hepato-intestinal or urogenital illnesses, collectively referred to as schistosomiasis^4^.

On the other hand, members of the genus *Trichobilharzia* infect birds as definitive hosts^2^. In water birds, *Trichobilharzia szidati* and *T. regenti* are key representatives that cause visceral and neurological forms of disease, respectively. Water birds release eggs containing larvae (miracidia) in the faeces (*T. szidati*) or miracidia from their nasal mucosa (*T. regenti*) into a freshwater environment. Here, motile miracidia seek out, find and infect a snail intermediate host, and undergo asexual reproduction, resulting in thousands of cercariae (invasive larvae) being shed into the water column. The cercariae find a definitive host (water bird) within 1 to 1.5 days^5^. Once they find this host, cercariae start to penetrate the skin using a cocktail of proteolytic enzymes (peptidases) and undergo morphological (metamorphosis) and molecular changes^6–8^. During penetration, cercariae lose their tail, shed their glycocalyx, form a tegumental double-membrane and switch from aerobic to facultative anaerobic metabolism^7,8^. Once inside the definitive host, transformed cercariae (schistosomula or schistosomules) of *T. szidati* and *T. regenti* use distinct migratory routes to establish infection in distinct tissues/organs and cause disease.

*Trichobilharzia szidati* is the species that causes visceral trichobilharziasis. The cercariae invade the avian host via skin, enter the circulatory system, and schistosomules reach the lungs within 2 days of infection and eventually develop to dioecious adults in the vessels of the intestinal wall, where they reproduce^9^. In the lungs, schistosomules can cause serious parasitic pneumonia, accompanied by lymphatic lesions and an influx of macrophages, heterophils and eosinophils, and/or death of the animal in severe cases^9^. Inside the host, *T. szidati* schistosomules evade the host’s immune response and ingest blood to acquire nutrients^10^, but the molecular basis for nutrient acquisition and their survival is unknown.

By contrast, *T. regenti* is the species that causes neural/nasal trichobilharziasis. Here, following cercarial penetration, schistosomules develop and migrate via the peripheral nerves, spinal cord and brain to the nasal mucosa, where the dioecious adults mate and reproduce. During their migration, schistosomules cause inflammation and neurodegenerative changes linked to motor neuronal malfunctions, and/or can lead to death of the animal, depending on the intensity of infection^11–13^. *Trichobilharzia regenti* schistosomules appear to feed on neural tissues rather than on blood^14^.

Despite of the differences in biology between *T. szidati* and *T. regenti*, and in the diseases that they cause, almost nothing is known at the molecular level about the difference(s) in their host-parasite relationship and the way in which their schistosomules feed, acquire nutrients and survive during the migration phase in the avian host. To date, most molecular biological studies have focused on human schistosomes^15–17^. This contrasts markedly the situation for other schistosomatids, for which the potential to explore transcriptomes, proteomes and gene function is only now being realised^18^. No nuclear genomic sequences have yet been published for *Trichobilharzia* species (except of draft genome of *T. regenti* (parasite.wormbase.org)), and transcriptomic and proteomic data are scant. However, a recent transcriptomic investigation provided a first glimpse of the molecular biology of development, adaptation and host invasion of *T. regenti*^18^. In the present study, were used previous^18^ and new data sets to compare the molecular basis of nutritional requirements of *T. regenti* and *T. szidati* schistosomules in their avian host.

## Results and Discussion

### Transcriptomes and homology-based comparisons

**Figure 1:**
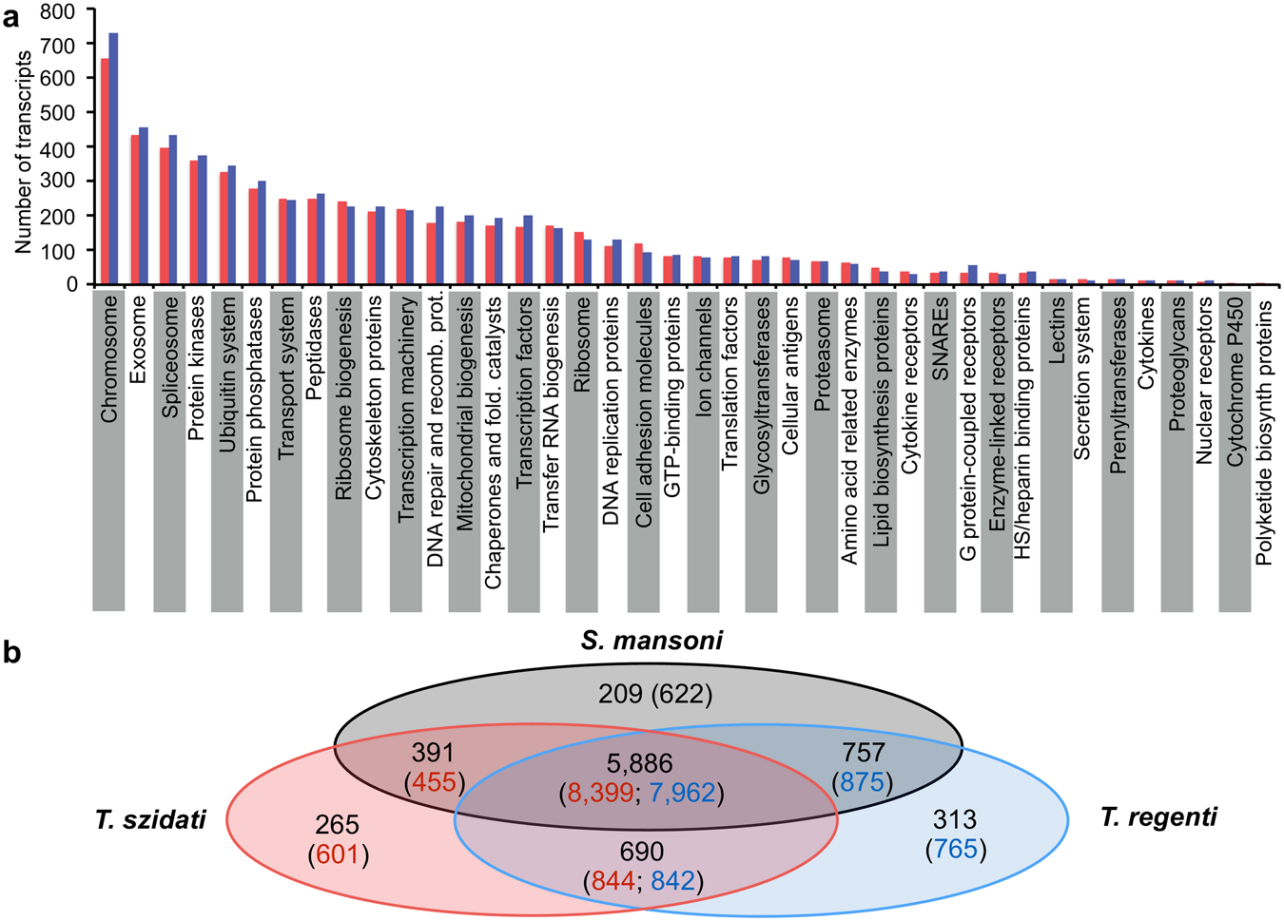
Annotation of protein-encoding genes transcribed in *Trichobilharzia szidati* and *T. regenti* larvae (cercaria combined with schistosomules) (**a**) Indicated are protein families represented in *T. szidati* (red) and *T. regenti* (blue) assigned *via* Kyoto Encyclopedia of Genes and Genomes (KEGG). Comparison of protein groups predicted from the larval transcriptomes among *T. szidati* and *T. regenti* and *Schistosoma mansoni* based on homology (OrthoMCL) (**b**) indicated are numbers of orthologous protein groups, followed by numbers of transcripts representing these groups (in brackets).

The transcriptomes of *T. szidati* (*n* = 13,007) and *T. regenti* (*n* = 12,705) contained similar numbers of transcripts and annotated transcripts (Table 1) and were 80.5% to 81.5% complete. In total, 728 of the BUSCOs in the transcriptomes of *T. szidati* (*n* = 787) and *T. regenti* (*n* = 797) were shared. Most (80%) transcripts in the transcriptomes of *T. szidati* (*n* = 10,498) and *T*. *regenti* (*n* = 11,114) shared amino acid sequence homology with proteins assigned one or more KEGG terms. Almost half of the transcripts (*n* = 5,663 and 5,935) associated with 3,065 and 3,275 KEGG terms with conserved protein family annotations (KEGG BRITE), respectively (Table 1), and 3,452 and 3,611 transcripts associated with 1,837 and 1,934 KEGG terms with KEGG biological pathway annotations, respectively (Fig. 1, panel a; Supplementary Table S1). In total, more than 460 excretory/secretory proteins were predicted from the transcriptomes of *T. szidati* (*n* = 642) and *T. regenti* (*n* = 468) (Supplementary Table S2).

**Table 1:** Characteristics of the *Trichobilharzia szidati* and *T. regenti* transcriptomes and annotation of their predicted proteomes.

	*T. szidati*	*T. regenti* ^18^
*Transcriptome*		
Total sequences	13,007	12,705
Minimum and maximum sequence lengths; N50 (bp)	160-39,264; 3,402	115-41,111; 3,682
*Predicted proteome*		
Total sequences	13,007	12,705
Minimum and maximum sequence lengths; N50 (bp)	30-11,108; 671	30-8,133; 805
*Matches to conserved metazoan BUSCO genes*		
Complete single copy orthologous groups^a^	603 (61.7)	659 (67.4)
Complete, duplicated orthologous groups^a^	97 (9.9)	81 (8.3)
Fragmented orthologous groups^a^	87 (8.9)	57 (5.8)
Total BUSCO orthologous groups^a^	978 (100)	978 (100)
*Protein annotation*		
NCBI nr database^b^	10,457 (80.4)	10,900 (85.8)
SwissProt^b^	7,633 (58.7)	8,120 (63.9)
MEROPS peptidases^b^	357 (2.7)	392 (3.1)
MEROPS inhibitors^b^	249 (1.9)	259 (2.0)
KEGG BRITE protein families^c^	5,663 (43.5; 3,065)	5,935 (46.7; 3,275)
KEGG pathway^c^	3,452 (26.5; 1,837)	3,611 (28.4; 1,934)
InterProScan^b^	8,171 (62.8)	9,642 (75.9)
Gene ontology annotation (InterProScan)^b^	6,730 (51.7)	6,961 (54.8)
*Proteins predicted to be excreted/secreted*	642 (4.9)	468 (3.7)

^a^Number of proteins homologous to BUSCO orthologous groups (% of total groups).

^b^Number of proteins homologous to entries in database (% of predicted proteome).

^c^Number of proteins homologous to entries in the KEGG database (% of predicted proteome; number of conserved KEGG terms).

An homology-based analysis showed that 5,886 orthologous protein groups were shared by *T. szidati*, *T. regenti* and *S. mansoni* (Fig. 1, panel b). Neurotropic *T. regenti* shared twice as many orthologous groups (*n* = 757) with *S. mansoni* than did visceral *T. szidati* (*n* = 391). Analyses of orthologues shared by *T. regenti* and *S. mansoni* revealed 145 transcripts assigned to “chromosome, DNA repair and recombination proteins, ubiquitin system, DNA replication proteins, protein kinases, chaperons and folding catalysts”; 112 transcripts of these orthologues were not detected in *T. szidati*, indicating differential transcription between species (upon pairwise comparison) rather than phylogenetic divergence. Transcripts that were unique to *T. szidati* (*n* = 601) and *T. regenti* (*n* = 765) linked to 20 and 18 unique protein families in the KEGG BRITE database, respectively, being assigned to one or more specific KEGG terms including spliceosome (7 for *T. szidati* and 11 for *T. regenti*), exosome (6 and 9), chromosome (5 and 9), peptidase (4 and 4) and kinase (2 and 6) (Supplementary Table S3).

### Differential transcription

**Figure 2:**
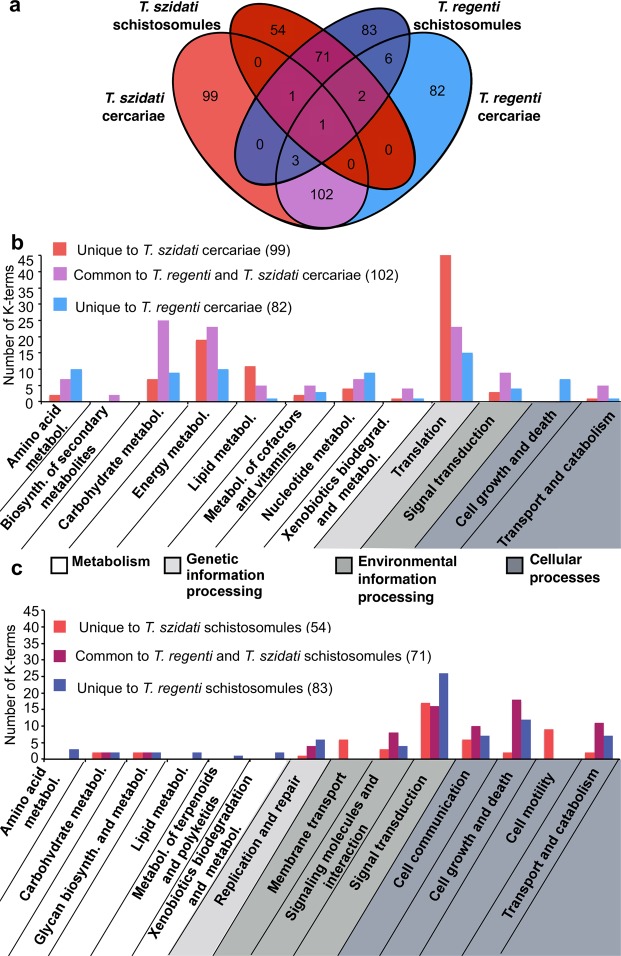
Comparison of enriched biological (KEGG) pathways common or specific to cercariae or schistosomules of *Trichobilharzia szidati* (red) and *T. regenti* (blue). Venn diagram-comparison of the numbers of K-terms that are common or specific to cercariae and/or schistosomules of *T. szidati* and/or *T. regenti* (**a**) Bar graph comparison of KEGG terms that are common or specific to either cercariae (**b**) or schistosomules (**c**).

**Figure 3:**
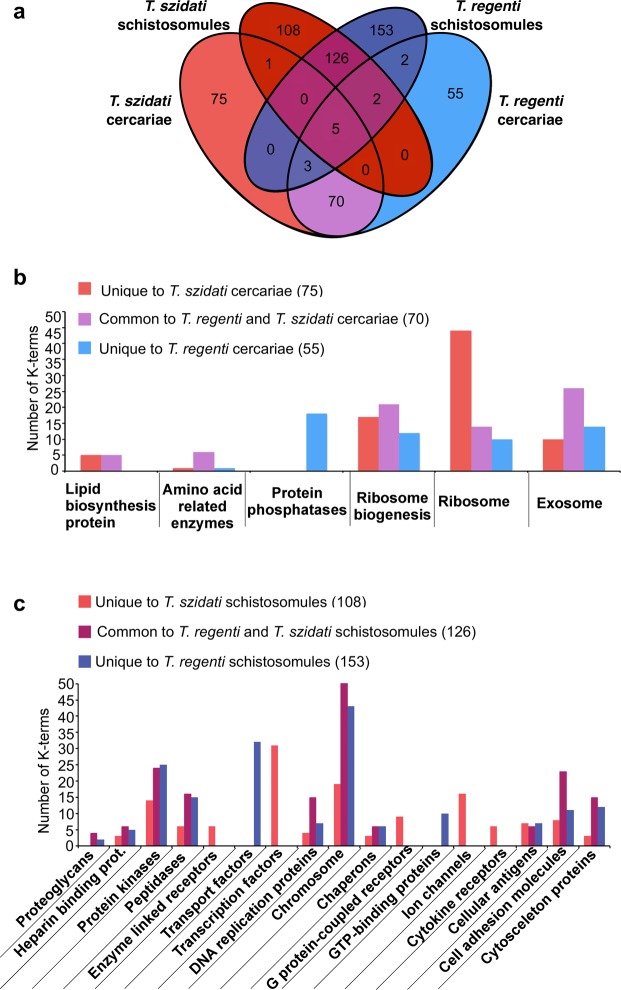
Comparison of enriched protein families (KEGG BRITE) common or specific to cercariae or schistsomules of *Trichobilharzia szidati* and *T. regenti*. Venn diagram-comparison of the numbers of KEGG terms that are common or specific to cercariae and/or schistosomules of *T. szidati* and/or *T. regenti* (**a**) Bar graph comparison of K-terms that are common or specific to either cercariae (**b**) or schistosomules (**c**).

Differential transcription was investigated between the free-living, infective (cercaria) and the migratory, parasitic (schistosomule) stages of *T. szidati* and *T. regenti*. In *T. szidati*, 3,729 of all 13,007 known transcripts were differentially transcribed between these two larval stages (1,881 up-regulated in the cercaria, and 1,848 up-regulated in the schistosomule). In *T. regenti*, 3,396 of all 12,705 known transcripts were differentially transcribed between these two stages (1,301 up-regulated in cercaria, and 1,876 up-regulated in schistosomule). For each species, differential transcripts were then linked to enriched biological pathways and protein families using the KEGG database, and the numbers of species- and stage-specific enriched biological pathways and protein families established (Figs 2 and 3).

### Pathways and protein families enriched in cercariae

A total of 283 KEGG pathway terms (99 for *T. szidati* and 82 for *T. regenti*; 102 shared by both) and 200 KEGG BRITE protein family terms (75 for *T. szidati* and 55 for *T. regenti*; 70 shared) were enriched (Figs 2 and 3) in the cercariae of both *Trichobilharzia* species. Compared with the schistosomules, the results showed that cercariae usually had greater enrichment for pathways or protein families associated with metabolism and translation. The former (i.e. metabolism) related predominantly to carbohydrate metabolism (linked to conserved pathways or processes, such as Krebs cycle, glycolysis, pentose phosphate pathway and pyruvate metabolism), and energy metabolism was reflected in an enrichment of proteins linked to oxidative phosphorylation. Enriched metabolic pathways linked specifically to Krebs cycle, glycolysis and oxidative phosphorylation reflect the aerobic metabolism of free-living cercariae, contrary to the microaerobic metabolism of schistosomules. The latter (i.e. translation) related predominantly to ribosomal proteins and proteins involved in ribosome biogenesis, proteins associated with exosomes, protein phosphatases, amino acid related proteins and lipid biogenesis, with *T. szidati* having a greater enrichment for ribosomal proteins than *T*. *regenti* (Supplementary Table S4).

### Pathways and protein families enriched in schistosomules

A total of 208 (54 for *T. szidati* and 83 for *T. regenti*; 71 shared by both) KEGG pathway terms and 387 (108 for *T. szidati* and 153 for *T. regenti*; 126 shared) KEGG BRITE protein family terms were enriched in the schistosomules of both species (Figs 2 and 3). The main biological pathways enriched in *T. regenti* and *T. szidati* schistosomules linked to signalling, cell growth and death, cell motility, transport and catabolism, membrane transport, and DNA replication and repair. A distinction between the two species was observed in an enrichment for signal transduction pathways (linked to Ras, MAPK, Rap1, Wnt and ErbB) that was exclusive to *T. regenti*. Enriched protein families were represented by chromosome-associated proteins, proteins linked with transcription, transport system, cell adhesion, cytoskeleton proteins, DNA replication, protein kinases and peptidases (Supplementary Table S4), and MEG-encoded secretory proteins and saposins were conspicuous.

### Microexon gene (MEG)-encoded secretory proteins

**Figure 4:**
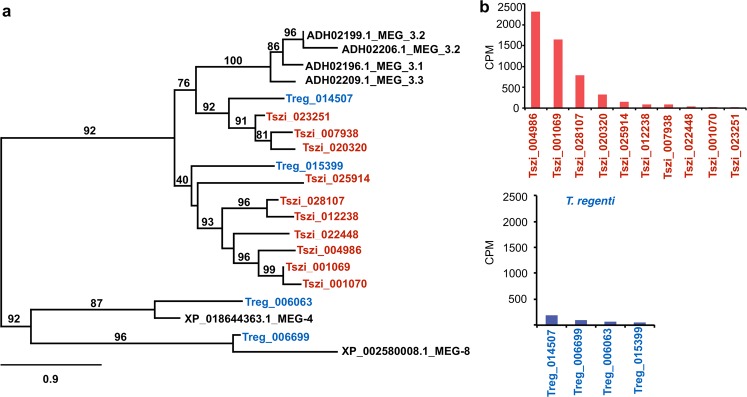
Phylogenetic relationships of protein sequences encoded by microexon genes (MEGs) among *Trichobilharzia szidati* (red), *T. regenti* (blue) and *Schistosoma mansoni* (reference taxon; black) constructed using a maximum likelihood (ML) tree building method, with nodal support values indicated (**a**), and a comparison of transcription levels (in counts per million reads, CPM) between *T. szidati* and *T. regenti* (**b**).

As MEG-encoded secretory proteins are proposed to be involved in blood processing in schistosomes^19,20^, we focused our attention on transcripts linked to this particular gene family in *T. szidati* and *T. regenti*. We detected 10 and 4 transcripts encoding MEGs in *T. szidati* and *T. regenti*, respectively. An analysis showed that predicted secretory proteins encoded by MEG orthologs of *T. szidati* and *T. regenti* clustered into four different groups (Fig. 4). *Trichobilharzia regenti* transcripts were represented in all four groups, suggesting that MEG-encoded secretory proteins are not strictly linked to visceral schistosomiasis^19^. While *S. mansoni* MEGs were assigned previously to three groups (MEG-3, MEG- 4 and MEG-8), some orthologs of *T. szidati* and *T. regenti* were not closely related to known MEGs of *S. mansoni* (Fig. 4), suggesting that they are specific to avian *Trichobilharzia* species. In *T. szidati*, MEG homologs were highly transcribed, with two of them (Tszi_001069 and Tszi_004986) being in the top-10 most upregulated transcripts in schistosomules. In *T. regenti*, MEG genes were transcribed at markedly lower levels than those in *T. szidati* (Fig. 4, panel b). Collectively, these results suggest that there is a differential involvement of MEG-encoded secretory proteins in feeding by the two *Trichobilharzia* species.

### Saposins

These lysosomal proteins can play a key role in the degradation of some sphingolipids^21^. Two transcripts (Treg_014592, Treg_015261) encoding saposin B were represented in the ten most upregulated transcripts in *T. regenti* schistosomules. Saposin B degrades sulfatides, a major component of the myelin sheaths of nerves^22^. The specific detection of myelin in the guts of *T. regenti* schistosomules suggests that saposin B is central to lipid degradation and digestion during their migration through the spinal cord. Saposin-encoding transcripts were also identified in *T. szidati* schistosomules, but at very low levels. A role for saposins in blood digestion has been proposed for *S. mansoni* and for the liver flukes *Clonorchis sinensis* and *Fasciola hepatica*^23–25^. Taken together, this information suggests that saposins linked to the degradation of macromolecular substrates, depending on the nutrition requirements of a particular fluke.

### Peptidases

**Figure 5:**
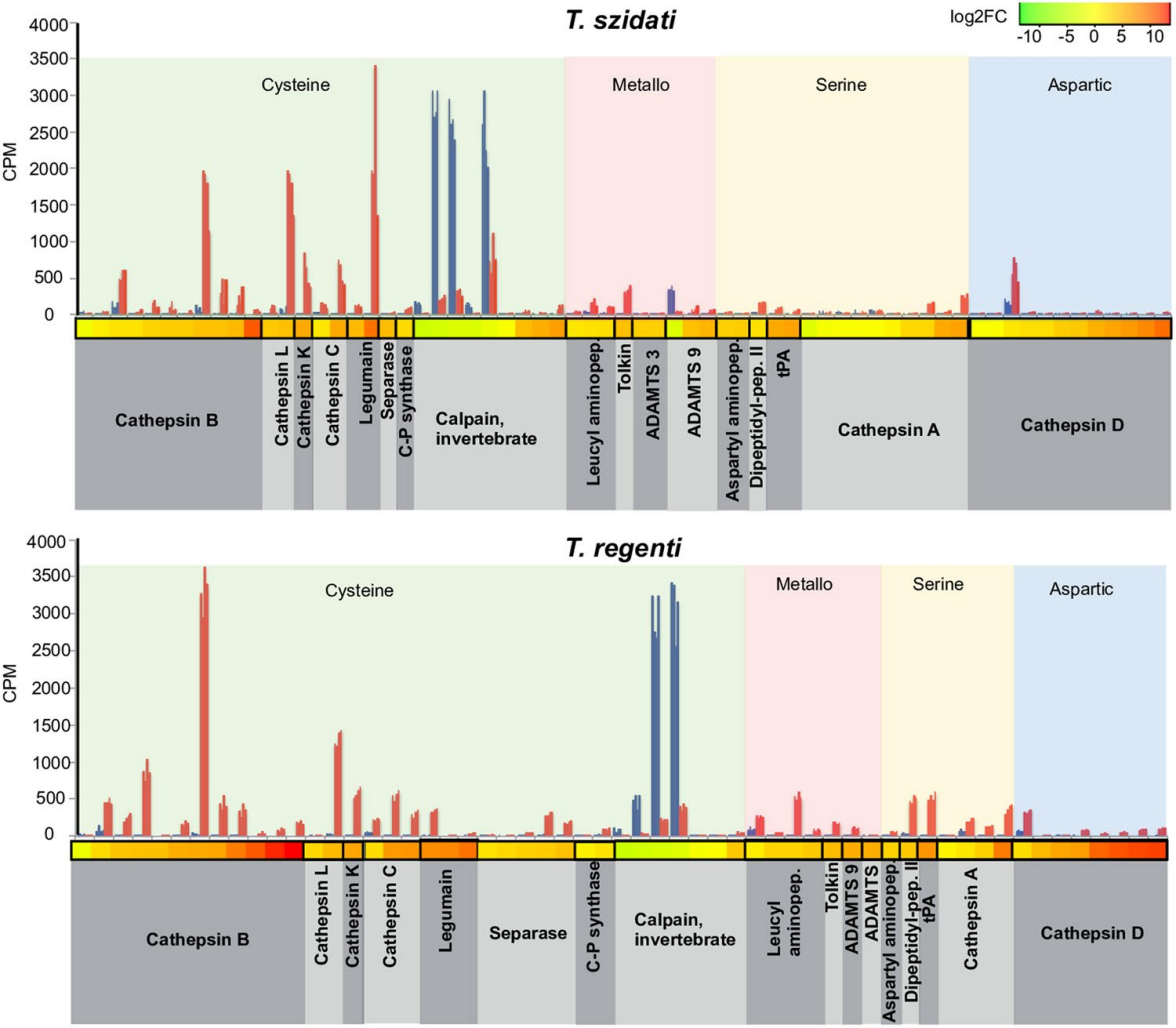
Transcription profiles of genes encoding peptidases (KEGG BRITE annotation) which were upregulated in cercariae (blue) or schistosomules (red) of *Trichobilharzia szidati* or *T. regenti*. Transcription levels, indicated by individual clusters of four biological replicates, are given in counts per million (CPM). Heat map colour indicates level of differential transcription between cercaria and schistosomule (log2-fold change).

Other evidence indicated a differing involvement of peptidases in the cercaria and schistosomule stages of *T. szidati* and *T. regenti*. Peptidases inferred to be enriched in the schistosomules of both species (Fig. 5; Supplementary Table S5) were principally the cathepsins (B, L, D, A, K and C), dipeptidyl-peptidase II, leucyl aminopeptidase, aspartyl peptidase, tissue plasminogen activator, legumain, calpain, tolkin, ADAMTS (a disintegrin and metalloproteinase with thrombospondin motifs) peptidases and carbamoyl-phosphate synthase. The transcription profiles representing these peptidases were similar between cercariae and schistosomules for both *Trichobilharzia* species (Fig. 5). Usually represented by relatively moderate to high numbers of transcripts (n > 7) were cathepsin B (11 for *T. szidati* and 12 for *T. regenti*), cathepsin D (11 and 8, respectively), cathepsin A (10 and 4, respectively) and calpain (9 and 7, respectively). Transcripts encoding calpain were abundant in both cercariae and schistosomules of both *T. szidati* and *T. regenti*, suggesting that this peptidase is multi-functional.

Following these analyses, we explored the abundance of transcripts encoding peptidases that were upregulated in the schistosomules of each species. In *T. szidati* schistosomules, transcripts encoding legumain were most abundant, followed equally by those encoding cathepsins L and B, followed by calpain, cathepsins D, K, C and A, leucyl aminopeptidase and dipeptidyl peptidase II (in order of decreasing abundance) (Fig. 5). In *T. regenti* schistosomules, transcripts representing cathepsin B were most abundant and twice as high as those representing cathepsin L, followed (in decreasing order) by transcripts representing cathepsins K and C, tissue plasminogen activator, leucyl aminopeptidase, dipeptidyl-peptidase II, cathepsin A, calpain and legumain. Transcripts representing selected peptidases were unique to *T. szidati* (i.e. disintegrin and metalloproteinase domain-containing protein 9) and to *T. regenti* (i.e. cathepsin S, caspase 7, disintegrin and metalloproteinase domain-containing protein 1 and anhydrolase domain-containing protein 5). These findings suggest that peptidases are the key enzymes involved the digestion and acquisition of nutrients from different macro-molecular substrates in schistosomules of *T. szidati* and *T. regenti*.

**Figure 6:**
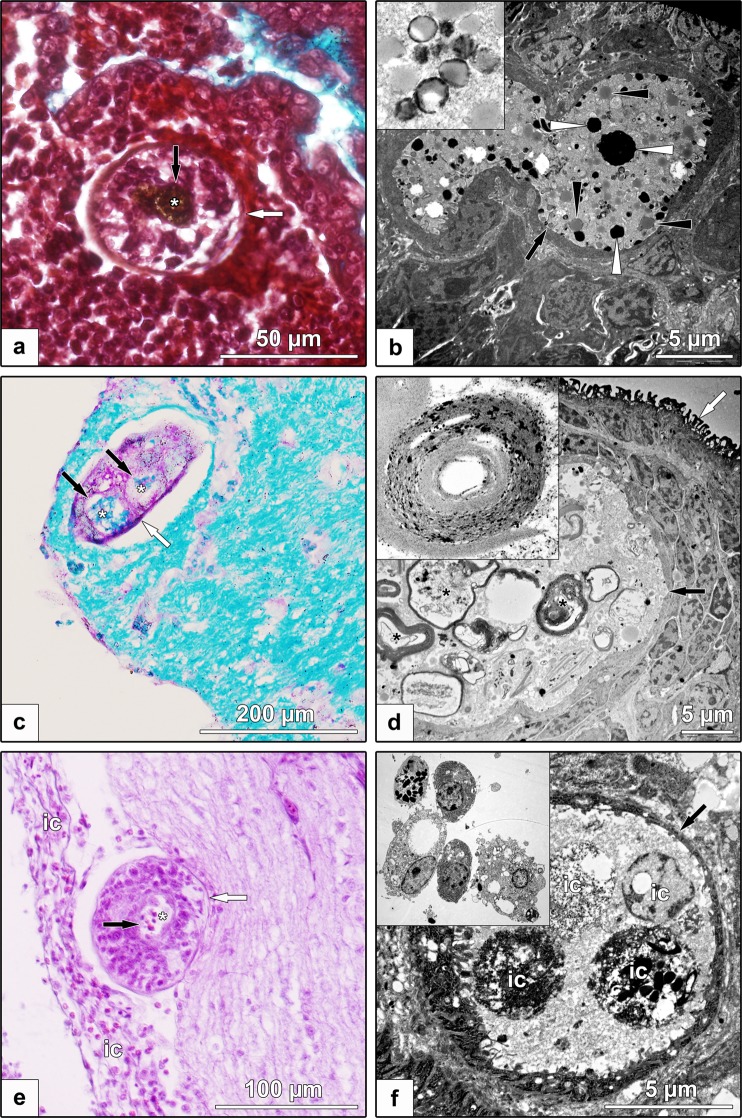
Light (left) and electron (right) microscopic examinations of schistosomules of *Trichobilharzia szidati* from the lungs and of *T. regenti* within the spinal cord from infected ducks. *T. szidati* schistosomules from/in lung (white arrow) with dark brown haem metabolites (white asterisk), crystals (white arrowheads) and/or lipid droplets (black arrowheads) in the gut lumen (black arrow). Detail of crystallization of hem metabolites is depicted in the box (**a**,**b**) *T. regenti* schistosomules (white arrow) migrating in the spinal cord with myelin (white asterisk) in the gut lumen (black arrows). Partially digested myelin sheath is depicted in detail in the box. (**c**,**d**) Immune cells surrounding (ic) or within the gut (black arrow, white asterisk) of *T. regenti* schistosomules (white arrow) in sub-meningeal space. Immune cells from sub-meningeal space are depicted in the box (**e** and **f**).

Although there were qualitative differences between the two species, the differential levels of transcription of genes encoding particular peptidases (taken as a snap-shot) indicate distinctive requirements of the schistosomules between the two species, to digest tissue types with differing macromolecular (protein) compositions (i.e. blood *versus* neural tissues). In particular, the high transcription of genes encoding cathepsins B and L and legumain (=haemoglobinase) are likely to be specifically linked to the degradation of blood^26,27^, which accords with microscopic evidence showing the presence of blood metabolites specifically in the gut of schistosomules of *T. szidati*, but not *T. regenti* (Fig. 6). By contrast, genes encoding legumain (haemoglobinase) were transcribed at a substantially lower level in schistosomules of *T. regenti* compared with those of *T. szidati*. As peptidases are often multi-functional^28^, it is possible that limiting amounts of legumain might activate (other) peptidases, such as cathepsin B, which is known to degrade myelin^29^ and is represented by the top transcribed peptidase gene (~3,300 CPM) in *T. regenti* (Fig. 5).

## Conclusion

Using a bioinformatics workflow, we explored the transcriptomic landscape of the cercarial and schistosomule stages of *T. szidati* and *T. regenti*, which assume distinct migratory paths and predilection sites in the avian host. This study allowed us to explore the differences in biology between these flukes before they enter (cercariae) and as they migrate (schistosomules) through tissues in the host animal and aspects likely linked to fluke-host cross-talk. The exploration indicated that both *T. szidati* and *T. regenti* schistosomules have key molecular pathways and repertoires of enzymes critical for acquisition and degradation of macromolecules (including proteins, lipoproteins and lipids) from distinct tissue types during their distinct migratory paths. Such repertoires likely allow these migratory flukes to effectively use key components in blood (*T. szidati*), nerves (*T. regenti*) and/or other tissues (both species) to migrate, develop and live in quite different physiological conditions.

Following skin penetration of the avian host, cercariae lose their tails and metamorphose to schistosomules which then migrate through tissues in the definitive host. In the penetration phase, cercariae of both species are enriched for pathways or molecules associated with carbohydrate metabolism (including glycolysis and Krebs cycle), oxidative phosphorylation and the translation of proteins including those required for ribosome biogenesis, exosome production and/or lipid biogenesis. In the migration phase, schistosomules of both species are enriched for pathways or molecules associated with processes including signal transduction, cell turnover and motility, DNA replication and repair, molecular transport and catabolism. The exclusive enrichment in signalling pathways linked to Ras, MAPK, Rap1, Wnt and ErbB in *T. regenti* appears to relate to processes including cell adhesion, proliferation, differentiation, migration, cell to cell communication and/or junction formation. These observations suggest the importance of cell adhesion molecules (CAMs) in this species^18^. CAMs are known to regulate host-parasite interactions, in addition to having crucial roles in the regulation of cellular integrity and interactions. The unique transcription profiles representing these molecules in schistosomules suggest an involvement in rapid growth and development of different organ structures in *T. regenti* within the avian host^30^ and/or host-parasite interactions. The former aspect is supported by an abundant transcription linked to neuroligin, netrin receptor and semaphorin in *T. regenti*^18^, which might relate to neural development in the schistosomule stage^31,32^.

In contrast to cercariae, which store and express proteolytic enzymes in their penetration glands for percutaneous invasion, schistosomules actively degrade various host tissue and cell types during migration^21^ and inhibit or evade immune attack by the host^33^. The markedly higher levels of transcription of MEG genes in *T. szidati* compared with *T. regenti* suggests variation in nutrient acquisition or parasite-host cross-talk between the two species of fluke. Although many peptidases in both species would have broad substrate specificity to degrade many proteins and lipoprotein complexes, selected peptidases likely have relative specificity to degrade blood (e.g., legumain) in *T. szidati* or myelin in nerves (e.g., cathepsin B) in *T. regenti*, reflecting an adaptation to distinct modes of nutrient acquisition between these visceral and neurotropic flukes of birds. The relatively high transcription of genes encoding a wide array of cathepsins and other proteolytic enzymes lends support for the involvement of this group of enzymes in numerous biological processes with key roles during the parasite’s migration, feeding and survival in the definitive host.

## Methods

### Ethics and study approval

Animal experimentation was approved (ref. no. MSMT-31114/2013-9 and MSMT-33740/2017-2) by Charles University and the Czech Ministry of Education, Youth and Sports, and was conducted in accordance with the European Directive 2010/63/EU and Czech Law for Biomedical Research (246/1992 and 359/2012).

### Parasite procurement

*Trichobilharzia szidati* and *T. regenti* were maintained in separate laboratories in snail intermediate (*Lymnaea stagnalis* or *Radix lagotis*) and definitive (*Anas platyrhynchos* f. *domestica*; breed - Cherry Valley strain) hosts using well-established protocols^8,18,34^. Life cycle stages of *T. szidati* were produced here, and those of *T. regenti* had been raised previously^18^. For each *Trichobilharzia* species, four independent biological replicates of cercariae were collected from distinct groups of infected snails (*n* = 20). For each replicate, 10,000 cercariae were washed extensively in H_2_0, pelleted by centrifugation at 2,500 *g* (4 °C), suspended in TRIzol (Thermo Fisher Scientific) and then frozen at −80 °C. For each *Trichobilharzia* species, four biological replicates of schistosomules were produced in (7-day old, helminth-free) ducks; each of four ducks was infected percutaneously with 2,500 cercariae^8,18,29^. Schistosomules were collected by microdissection from the lungs (*T. szidati*) and spinal cord (*T. regenti*) at 90 h and 162 h following infection of ducks, respectively; these time points were selected to ensure that the development of the schistosomules of the two species (collected from the distinct tissues) was the same and thus comparable^2,35,36^. For each replicate, 300 schistosomules were washed extensively in phosphate-buffered saline (PBS, pH 7.0), pelleted, suspended in TRIzol and frozen in the same manner as for cercariae.

### RNA-sequencing

RNAs of *T. szidati* were sequenced here, and those of *T. regenti* had been done previously^18^. For both *T. szidati* and *T. regenti*, total RNA was isolated from each of the four replicates of each cercariae and schistosomules and *DN*ase I-treated^18^. RNA quality was assessed using a Bioanalyser 2100 (Agilent), and the quantity was estimated using a Qubit 4 fluorometer (Invitrogen). From each replicate representing each species and developmental stage of *Trichobilharzia*, mRNAs were isolated to construct short-insert (330 bp) cDNA libraries, barcoded according to the manufacturer’s instructions (TruSeq RNASamplePreparation v.2, Illumina). For each species, all eight cDNA libraries were each pair-end sequenced (2 × 211 bp reads) using the HiSeq. 2500 platform (Illumina)^18^.

### Transcriptome assembly and protein prediction

An established bioinformatic workflow system^18^ was used for assembly. In brief, adaptors and nucleotides with Phred quality scores of ≤20 were removed from raw RNA-seq reads from each library using Trimmomatic^37^ v.0.3. Reads were error-corrected using BayesHammer in the software package SPAdes^38^ v.3.1.0 and normalised using khmer^39^ v.1.1. Read datasets were mapped specifically to the duck genome (BioProject no. PRJNA46621) using TopHat^40^ v.2.1.1 and host sequences removed.

For each *Trichobilharzia* species, RNA-seq datasets representing the cercariae and schistosomules were pooled and employed to assemble a non-redundant transcriptome using Oases^41^ v.0.2.8. In order to achieve an optimum transcriptome for each species and each replicate, various *k*-mer (21 to 49) and coverage (5 to 21) threshold values were assessed during the assembly process. Based on number of contigs, length of contigs, and transcript redundancy, the best assemblies were achieved using *k*-mers of 47 to 49 and coverages of 9 to 14. For each species, the eight transcriptomes representing the individual replicates of each of the larval stages (cercaria and schistosomule) were pooled and any redundancy removed using CD-HIT-EST^42^ employing a nucleotide identity threshold of 85%. Protein-coding regions were predicted from individual non-redundant transcripts using Transdecoder^43^ v.3.0.0 employing a minimum length of 30 amino acids (aa).

### Transcriptome annotation and curation

An established pipeline^44^ was used for annotation. Predicted protein sequences were annotated using their closest homologues (BLASTp; E-value cut-off: ≤10^−5^) employing the NCBI non-redundant protein, Kyoto Encyclopedia of Genes and Genomes (KEGG), excluding the “organismal system” and “human disease” categories^45^, and UniProt^46^. The proteomes predicted separately from the larval transcriptomes of *T. szidati* and *T. regenti* were compared with that of *S. mansoni*^47^. For each *Trichobilharzia* species, orthologous and unique protein groups were identified using OrthoMCL^48^ (E-value cut-off: ≤10^−5^; similarity cut-off: 0.5). The completeness of each transcriptome was assessed by *b*enchmarking *u*niversal *s*ingle-*c*opy *o*rthologs (BUSCO)^49^. Excretory/secretory proteins were predicted using the prokaryotic and eukaryotic classical analysis of secretomes (PECAS) pipeline employing default settings^50^.

Subsequently, each transcriptome was curated. Transcripts with high nucleotide identity (BLASTn; E-value: ≤10^−5^) to avian, bacterial or viral sequences present in the NCBI non-redundant nucleotide database (NCBI) were removed. Any conceptually translated protein with a high amino acid sequence homology (BLASTp; E-value cut-off: ≤10^−5^) to transposable elements in RepBase database^51^ were also eliminated, as were translated sequences of ≤50 amino acids with no homology (at the nucleotide and protein levels) to sequences in current public databases. Additionally, transcripts with read counts of <10 (using RSEM)^52^ in all replicates of individual developmental stages were removed from each assembled transcriptome.

Microexon gene (MEG) transcripts were annotated by comparison (BLASTp; E-value: ≤ 10^−5^) with reference sequences in the GenBank database (accession nos. ADH02196.1, ADH02199.1, ADH02206.1, ADH02209.1, XP_018644363.1, XP_002580008.1). Amino acid sequences inferred from these transcripts were aligned using the program MUSCLE^53^ v 3.8.31 and maximum likelihood (ML) phylogenetic tree built employing PhyML^54^ v 3.1 using a WAG model of amino acids substitution and a shape parameter of gamma distribution of 3.758; the resultant tree was displayed using TreeDyn^55^.

### Differential transcription and enrichment analyses

An established method^18^ was used for this analysis. For each *Trichobilharzia* species and each replicate, the trimmed, corrected, paired reads were mapped to the respective non-redundant, merged transcriptome using RSEM^52^. Differential gene transcription between cercariae and schistosomules was calculated using edgeR^56^ v.3.6.7 and R^57^ 3.2.3. Read counts (in counts per million, CPM) were normalised for G+C bias^58^ and for the trimmed mean of M-values (TMM)^59^. The edgeR exactTest function was used to assess differential transcription. The multiplicity correction of *P*-values was then performed by applying the Benjamini Hochberg method to establish the false discovery rate. Transcripts were considered differentially transcribed if the latter rate was ≤0.01. For differentially transcribed molecules, enriched metabolic pathways and protein families were identified using the KEGG database^40^. Upregulated transcripts assigned to KEGG orthology (KO) terms were mapped to KEGG pathways and the KEGG BRITE database. A Fisher’s exact test was used to calculate levels of statistical significance in differences between numbers of KO terms assigned to particular pathway/enzyme classes; a *P-*value of ≤0.05 was set as the threshold for significant enrichment in a particular developmental stage of each *Trichobilharzia* species studied.

### Microscopic examinations

In the light microscopic examination, tissues containing schistosomules were collected from ducks infected for 4 days with *T. szidati* (lungs) or for 7 days with *T. regenti* (spinal cord) and immediately fixed in Bouin’s solution (cat. no. HT10132, Sigma-Aldrich). These tissues were processed and stained with Masson’s trichrome (lungs), haematoxylin-eosin or with Luxol fast blue (spinal cord) for histological examination using standard procedures. Sections were examined using a BX51 microscope (Olympus), and photographically documented using a DP72 camera (Olympus) and software Quick Photo Micro 3.1. (Promicra).

In the transmission electron microscopic examination, schistosomules of *T. szidati* (4 days) dissected from lung tissues and those of *T. regenti* (10 days) within spinal cord tissue blocks (4–6 mm^3^) were immediately fixed in modified Karnovsky solution^60^, fixed in OsO_4_ and then embedded in Spurr resin (SPI-supplies) using established protocols^61^. Schistosomules and/or tissues were subjected to transmission electron microscopy (JEOL JEM-1011) and photographed using a CCD camera (Veleta) employing acquisition software (Olympus Soft Imaging Solution GmbH).

## Supplementary information

Supplementary Table S1

Supplementary Table S2

Supplementary Table S3

Supplementary Table S4

Supplementary Table S5

## Data Availability

Short sequence reads and transcriptome analysed are available via Bioproject Submission SUB4374991.
